# Hyperspectral cell sociology reveals spatial tumor-immune cell interactions associated with lung cancer recurrence

**DOI:** 10.1186/s40425-018-0488-6

**Published:** 2019-01-16

**Authors:** Katey S. S. Enfield, Spencer D. Martin, Erin A. Marshall, Sonia H. Y. Kung, Paul Gallagher, Katy Milne, Zhaoyang Chen, Brad H. Nelson, Stephen Lam, John C. English, Calum E. MacAulay, Wan L. Lam, Martial Guillaud

**Affiliations:** 1 Integrative Oncology, British Columbia Cancer Research Centre, Vancouver, BC V5Z1L3 Canada; 2 Faculty of Medicine, University of British Columbia, Vancouver, BC Canada; 3 Deeley Research Centre, Victoria, BC Canada; 4 Pathology and Laboratory Medicine, Vancouver Coastal Health, Vancouver, BC Canada; 5 Department of Medical Genetics, University of British Columbia, Vancouver, BC Canada; 6 Department of Pathology and Laboratory Medicine, University of British Columbia, Vancouver, BC Canada

**Keywords:** Cell sociology, Hyperspectral imaging, Lung cancer, Immunohistochemistry, T cell, Lung cancer, CD3, CD8, CD79a, Spatial organization

## Abstract

**Background:**

The tumor microenvironment (TME) is a complex mixture of tumor epithelium, stroma and immune cells, and the immune component of the TME is highly prognostic for tumor progression and patient outcome. In lung cancer, anti-PD-1 therapy significantly improves patient survival through activation of T cell cytotoxicity against tumor cells. Direct contact between CD8+ T cells and target cells is necessary for CD8+ T cell activity, indicating that spatial organization of immune cells within the TME reflects a critical process in anti-tumor immunity. Current immunohistochemistry (IHC) imaging techniques identify immune cell numbers and densities, but lack assessment of cell–cell spatial relationships (or “cell sociology”). Immune functionality, however, is often dictated by cell-to-cell contact and cannot be resolved by simple metrics of cell density (for example, number of cells per mm^2^). To address this issue, we developed a Hyperspectral Cell Sociology technology platform for the analysis of cell–cell interactions in multi-channel IHC-stained tissue.

**Methods:**

Tissue sections of primary tumors from lung adenocarcinoma patients with known clinical outcome were stained using multiplex IHC for CD3, CD8, and CD79a, and hyperspectral image analysis determined the phenotype of all cells. A Voronoi diagram for each cell was used to approximate cell boundaries, and the cell type of all neighboring cells was identified and quantified. Monte Carlo analysis was used to assess whether cell sociology patterns were likely due to random distributions of the cells.

**Results:**

High density of intra-tumoral CD8+ T cells was significantly associated with non-recurrence of tumors. A cell sociology pattern of CD8+ T cells surrounded by tumor cells was more significantly associated with non-recurrence compared to CD8+ T cell density alone. CD3+ CD8- T cells surrounded by tumor cells was also associated with non-recurrence, but at a similar significance as cell density alone. Cell sociology metrics improved recurrence classifications of 12 patients. Monte Carlo re-sampling analysis determined that these cell sociology patterns were non-random.

**Conclusion:**

Hyperspectral Cell Sociology expands our understanding of the complex interplay between tumor cells and immune infiltrate. This technology could improve predictions of responses to immunotherapy and lead to a deeper understanding of anti-tumor immunity.

**Electronic supplementary material:**

The online version of this article (10.1186/s40425-018-0488-6) contains supplementary material, which is available to authorized users.

## Background

Information obtained from formalin fixed paraffin embedded (FFPE) tissues is crucial for clinical management of cancer patients. Standard of care includes routine immunohistochemistry (IHC) staining of FFPE sections for cancer diagnosis, prognosis, and for guiding choice of therapy [1]. Increasingly, clinical research groups are transitioning towards multicolor antibody panels to detect more complex cell phenotypes from a single slide [2]. While this approach offers additional biological information, multicolor staining requires specialized equipment and analysis software. Recently, commercially available multispectral imaging and analysis systems have been developed that can quantify cell types based on target protein expression and assess their localization within epithelial and stromal compartments [3].

These technologies have been instrumental in the advancement of cancer immunology as different immune markers have both prognostic and predictive power. For example, the presence of intratumoral T cells has been associated with improved patient outcomes in multiple cancer types [4–6], leading to increased focus on enhancing the activity of these cells. In the therapeutic setting, some studies have found that PD-L1 staining was associated with improved response to immune checkpoint blockade antibodies [7], which are thought to reverse T cell exhaustion. However, inconsistencies in these studies may be due to the acknowledged highly heterogenous staining of PD-L1, rendering IHC staining difficult to interpret [8–11]. We were intrigued by the possibility that a more detailed analysis relating the spatial relationships of specific immune cell subtypes with tumor cells and stroma could enhance the value of IHC in the context of prognostic and predictive biomarker staining.

Methods to understand the complex biology and the prognostic and predictive implications of the many cell types within the tumor microenvironment (TME) are rapidly progressing. Techniques to incorporate spatial information with hyperspectral analysis typically involve identifying specific compartments within the TME [3, 12, 13]. While these methodologies identify clinically relevant compartments and cell densities within them, they are currently severely limited in their ability to reproducibly assess, in an automated fashion, all aspects of tumor architecture and the associated immune cell population. Development of methods to investigate these cell-cell spatial relationships is crucial for advancing our understanding of tumor-immune interactions and it is anticipated that this form of analysis will generate a higher-order of useful information that cannot be provided by crude measures of cell density and distribution.

We utilize the term “cell sociology” to conceptualize the morphological observation of adjacency and contiguity between selected target cell populations. This incorporates the premise that direct cell-cell communication is one integral part of the biological process of anti-tumor immunity, exemplified by the complex network of interactions between antigen presenting cells, T cells, and target cells. For example, interactions between CD4+ T cells and CD8+ T cells help determine the level of anti-tumor immunity [14], whereas the interaction between CD8+ PD1+ T cells and PD-L1+ target cells can lead to T cell exhaustion and reduction, if not outright abrogation, of anti-tumor activity [15]. Importantly, quantification of cell sociology has the potential to afford greater prognostic and/or predictive insight on host anti-tumor immunity than has historically been provided by cell density evaluation alone.

To this end, we developed the Hyperspectral Cell Sociology platform. This technology images multiplex IHC-stained tissue, quantifies cell subsets, and measures spatial relationships between cell types of interest. The multispectral detector component of this image analysis sytem exploits the ability to detect IHC-linked chromagens that may be difficult to resolve by human microscopic observation due to different (often faint) densities in staining or due to superimposition of multiple-stained structures. This results in a detailed map of cellular topography of any selected tumor area. The individual elements of the map may then be quantified and related in a spatial paradigm. As a proof of concept, we illustrate the cell sociology of CD3+ T cells, CD3+ CD8+ T cells, CD79a+ B cells, and unstained cells in full tissue sections of lung adenocarcinoma, revealing novel parameters that are important for anti-cancer immunity.

## Methods

### Patient tissue accrual

*British Columbia Cancer Agency*. FFPE tumor tissues (*n* = 20) were obtained from the Tumor Tissue Repository of the British Columbia Cancer Agency or Vancouver General Hospital under informed written patient consent and with approval from the University of British Columbia – BC Cancer Agency (BCCA) Research Ethics Board (Table 1).

**Table 1:** Clinical features of lung adenocarcinoma cases. Patient clinical information and tumor characteristics for recurrent and non-recurrent lung adenocarcinoma cases (*n* = 20)

Patient ID	Recurrence	Recurrence site	Predominant Histological Pattern	Age	Sex	T	N	M	Max. Dimension (cm)	Stage	Adjuvant chemo-therapy^a^	Adjuvant radiation	Smoking	Mutation	Survival (Months)
P1	NA		Acinar	71	F	1	0	0	2.8	IA	No	No	CS	KRAS	96
P2	No		Acinar	82	F	2	0	0	3	IB	No	No	EX	EGFR	81
P3			Acinar	68	F	1	0	0	1.8	IA	No	No	NS	WT	77
P4			Acinar	57	F	2	1	0	2.8	IIB	Yes	No	CS	KRAS	86
P5			Acinar	67	F	2	1	0	3.2	IIB	Yes	No	CS	WT	88
P6			Acinar	71	F	2	0	0	3.1	IB	No	No	NS	WT	26
P7			Acinar	70	M	1	0	0	2.3	IA	No	No	CS	NA	95
P8			Mucinous	86	F	2	0	0	3.2	IB	No	No	NS	KRAS	92
P9			Acinar	82	F	3	0	0	2.7	IIB	No	No	NS	EGFR	98
P10	Yes	distant	Acinar	73	F	4	1	0	2.8^b^	IIA	No	No	CS	NA	63
P11		distant	Acinar	77	F	2	0	0	3.4	IB	No	No	NS	WT	13
P12		distant	Papillary	90	F	2	1	0	4.5	IIB	No	No	EX	WT	30
P13		local	Mucinous	77	F	1	0	0	2.5	IA	No	No	NS	KRAS	76
P14		distant	Acinar	53	F	2	2	0	7	IIIA	No	No	CS	KRAS	3
P15		local and distant	Papillary	60	F	2	1	0	2.5	IIB	Yes	No	EX	EGFR	77
P16		local and distant	Acinar	72	F	2	0	0	4.5	IB	No	No	CS	WT	61
P17		distant	Acinar	58	M	2	1	0	2.5	IIB	No	No	EX	WT	30
P18		distant	Micro-papillary	66	M	2	2	0	3.5	IIIA	Yes	No	NS	EGFR	58
P19		distant	Papillary	77	F	2	1	0	4	IIB	No	No	NS	EGFR	46
P20		distant	Acinar	78	F	1	0	0	1.2	IA	No	No	CS	KRAS	37

*M* Male, *F* Female, *CS* Current Smoker, *EX* Former Smoker, *NS* Never Smoker

^a^Adjuvant chemotherapy: cisplatin-vinorelbine, 4 cycles except for patient P4 who received 3 cycles of cyclophosphamide, doxorubicin and was then on tamoxifen because of synchronous breast cancer

^b^plus four additional carcinomas removed, giving the final T4 classification

### Multicolor immunohistochemistry

**Fig. 1:**
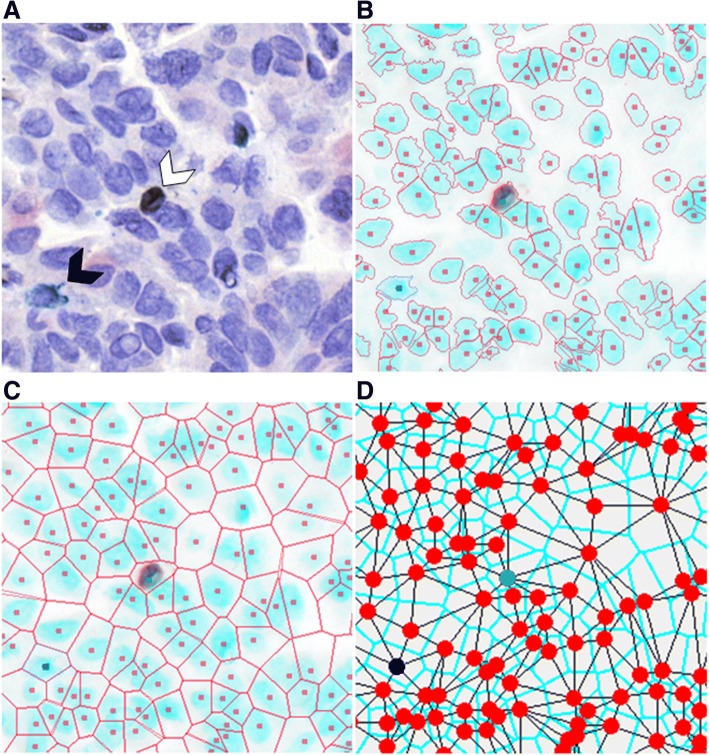
Hyperspectral imaging of multicolor immunohistochemistry. **a** Example of a lung tumor section that was stained with multicolor immunohistochemistry for CD3 (blue, black arrow), CD8 (dark brown, white arrow), CD79a (red) and counterstained with haematoxylin (nuclear stain) and imaged in white light. **b** Following hyperspectral imaging and spectral unmixing, nuclei were identified using the haematoxylin spectrum and are shown in blue. Nuclear boundaries were located using an edge location algorithm and are shown as red lines. **c** Voronoi diagrams were generated for each segmented cell and shown as red lines with dots as centers of cells. Cell neighbors are cell pairs that share a Voronoi edge. **d** The interaction between each neighbor is indicated by a black line connecting nuclei centres. In panels B-D, unstained cells are represented by a red centre, CD3+ T cells are represented by a blue centre, and CD3+ CD8+ T cells are represented by a green centre

FFPE tumor tissues were sectioned to 4 μm, baked at 37 °C overnight, and deparaffinized. Antigen retrieval was performed using Decloaking Chamber Plus with Diva decloaker (Biocare). Sections were blocked with peroxidazed-1 and background sniper 1. Slides were then stained with an anti-CD3 (SP7/Spring Biosciences)/ anti-CD8 (C8/144B/Sigma-Aldrich) cocktail diluted in DaVinci Green diluent, Mach2 Double Stain #2, IP Ferangi Blue, and IP DAB within an Intellipath FLX rack. Slides were rinsed with dH_2_O, incubated in SDS-glycine pH 2.0 for 45 min at 50 °C, and rinsed with dH_2_O [16]. Slides were then stained with Mouse AP polymer, Warp Red chromogen, and CAT hematoxylin counter stain. Second round staining included anti-CD79a (SP18/Spring Biosciences), DaVinci Green diluent, Mach 2 Mouse-AP polymer, IP Warp Red chromogen, and 1:5 dilution of CAT hematoxylin counterstain [16]. White light images were analyzed using a Pannoramic Digital Slide Scanner from 3D Histotech and the Pannoramic Viewer software (v1.15). A representative image is shown in Fig. 1a.

### Hyperspectral whole slide imaging system

Based on direction from a pulmonary pathologist (JCE), five areas (8000 × 8000 pixels) were investigated from each of 20 whole tissue sections. We developed an in-house automated hyperspectral whole slide imaging system that consisted of a computer controlled x-y stage (Marzhauser Wetzlar SCAN series), a Zeiss Axioscope 2 Mot plus microscope, a CRI Varispec tunable light filter, an Andor Neo (2 K by 2 K) camera, and a Zeiss 20X (NA 0.75) plan APROCHROMAT objective lens. We acquired images with a pixel sampling spacing of 0.33 μm (20X magnification). The user selected the area to be imaged on the slides and the absorption spectra of the labels (antibodies stain color). Based upon previously recorded stain spectra, the system automatically selected wavelengths to be imaged and collected (using a continuously active focus) up to 16 different spectral images per camera field at 20 nm increments (420 nm–720 nm). Spectral images from a blank area of each of the slides were used for calibration purposes, and each spectral image was corrected for illumination intensity and uniformity, filter efficiency, and camera fixed pattern noise. Once the area was imaged, the system stitched all overlapping camera fields together to make a seamless hyperspectral image. Regions of interest (ROI) of each image were manually generated, under direction of the pathologist, to encircle predominantly epithelial tumor cell containing areas. This excluded tissue artefacts or blank space that could have confounded downstream analysis.

### Spectral unmixing

Using the absorption spectra of each antibody and haematoxylin, the stains were computationally unmixed to determine the concentration of each stain for every pixel in the selected area (Additional file 1: Figure S1). The program assumed that every pixel in the flat field and dark field corrected recorded images (16 wavelegths) were a linear combination of the concentration of the individual stains occurring at that pixel weighted by the absorption characteristics of each of the stains occurring at that pixel. When mathematically separating the components, the program added an addition error term to each pixel to compensate for the electronic and photonic noise in the images. To separate these linear combinations of absorption stains with differing concentration at each pixel, the Multivariate Curve Resolution – Alternating Least Squares algorithm was used (MATLAB R2014a). For this process, the collected hyperspectral image data was log10 transformed and modeled to consist of 4 concentration images (one per stain used) multiplied by the spectra of each stain plus an error term [17, 18]. To use this method, the following were assumed throughout the analysis: (1) the spectra were constant; (2) there were no negative concentrations; and (3) the concentration at each pixel was not required to add to one (denoted as additivity). These assumptions removed the requirement of prior knowledge of the absorption spectra of the glass, stains and tissue, which would be required if the additivity constraint was used.

### Image segmentation

Each image was visualized and evaluated for sample quality; areas with a large proportion of empty space were discarded, leaving a total of 94 areas for further analysis. Automatic thresholding for stain positivity and nuclei segmentation were performed as previously described [19] and customized for each image to adjust for variability in the tissue architecture, cellular composition, and stain intensity. To identify individual nuclei, the unmixed haematoxylin image was segmented using multiple iterations of increasing stain intensity thresholds with the Otsu algorithm. The first iteration removed small objects (< 50 pixels) and classified large objects (> 1000 pixels) for additional segmentation iterations. Subsequent iterations used increasing intensity thresholds resulting in increasing segmentation of nuclei. Segmented objects that were > 150 pixels were considered as new separate objects, and segmented objects < 150 pixels were considered part of the original object and reattached to it. After each iteration, segmented objects were processed by a distance transform and watershed algorithms to determine whether the objects required additional segmentation, and objects > 1000 pixels were reprocessed with further, more strigent segmentation (higher thresholds), for up to three iterations. Finally, an edge relocation algorithm refined the nuclei boundaries [20] (Fig. 1b).

### Multicolor stain characteristics of segmented cells

The intensity threshold of each stain within the boundary of segmented nuclei was manually adjusted to differentiate stain positivity from background. Segmented cells were visualized in order of decreasing concentration of the selected antibody intensity, and the user determined the intensity threshold at which true positive cells were differentiated from background (Additional file 2: Figure S2). Thresholds were customized for each stain on each slide and applied across all areas imaged from the same slide. A three level binary decision tree was constructed to categorize each cell into a group based on the positivity or negativity of each stain, resulting in eight (2^3^) possible groups (Additional file 3: Figure S3A). During classification, each cell that was assigned incompatible markers was assessed manually (for example CD3, a T cell marker, co-stained with CD79a, a B cell marker). The majority of double (CD3+ CD79a+) or triple (CD3+ CD8+ CD79a+) positive-stained cells were overlapping T and B cells that could not be differentiated by the segmentation algorithm. Cells within groups corresponding to overlapping T and B cells were processed such that a nucleus was automatically added beside the existing nucleus, and one nucleus was assigned as a T cell and one as a B cell (Additional file 3: Figure S3B). However, when evidence of stain positivity of incompatible markers was observed in a single cell (and not in overlapping cells), the cells were excluded from analysis (Additional file 3: Figure S3C). Cells categorized as CD3-CD8+ CD79a+ and CD3-CD8+ were rare and were assigned to the unstained group to preserve the tissue architecture. Most cells in this category were staining artifacts, though some CD3-CD8+ cells may have been CD8+ natural killer cells.

### Quantification of cell sociology

The coordinates of cell nuclei were used to build Voronoi diagrams [21]. The Voronoi diagram represented a simple geometric and topological model of epithelial tissues, from which spatial relationships and neighborhood information could be extracted [22]. A neighborhood was defined by the user, and can be conceptualized as a neighborhood in a city. In this analogy, cells in a tissue sample could be considered as houses in a neighborhood, and two adjacent cells would be next-door neighbors. Cell neighbors were identified by first associating a Voronoi polygon to each nucleus, whereby the boundary between cells was positioned at the geometric mean between cell nuclei centers. The Voronoi polygon served as a good approximation of the cell membrane location (Fig. 1c). Cell-neighbor information was calculated for every cell in an imaged area (Fig. 1d), and these specific relationships were used to examine the cell sociology in each tumor.

**Fig. 2:**
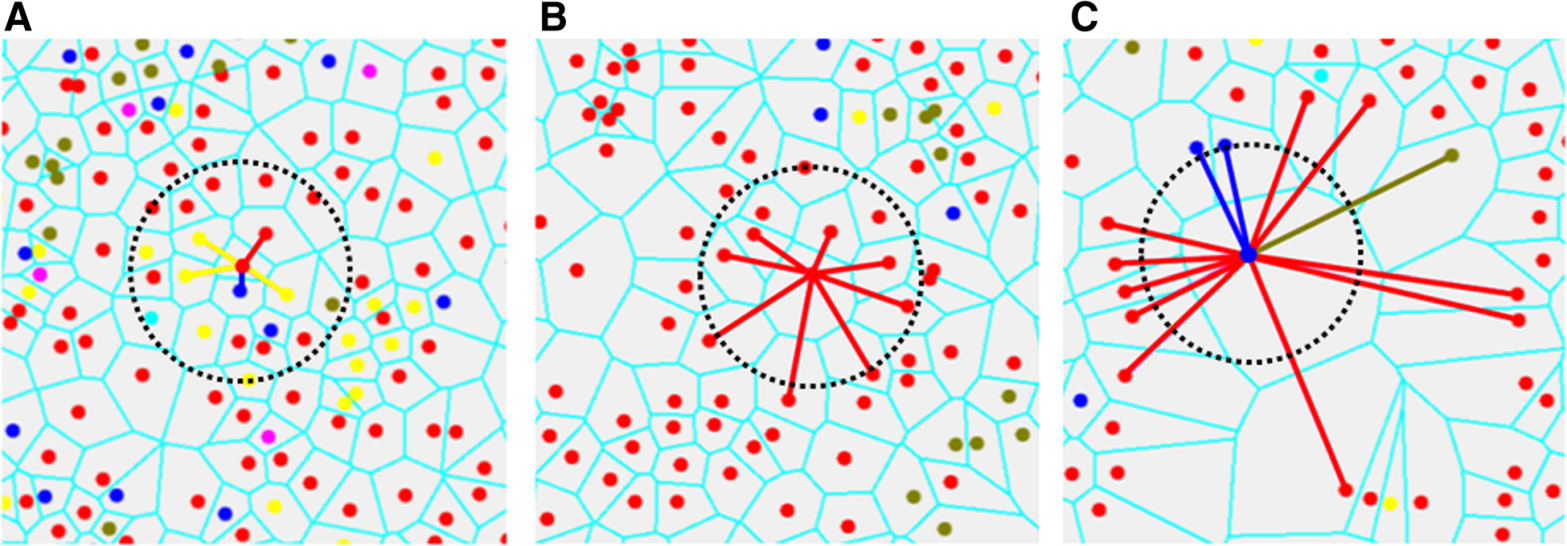
Quantification of cell sociology. Three examples where the neighbor fractions of a single cell per image is shown. Lines represent cell – cell interactions between neighbors to the central cell and the color of the lines represent the color of the neighbor cell. For cell sociology, all cells in the image would be analyzed in the same manner to determine average neighbor frequencies of each cell type. **a** An example of a red cell with five neighbors (cells that share a common Voronoi edge) is shown: one red neighbor (1/5), one blue neighbor (1/5), and three yellow neighbors (3/5). For the red cell in the centre, the neighbor frequency of red cells is 0.2, the neighbor frequency of blue cells is 0.2, and the neighbor frequency of yellow cells is 0.6. **b** An example of a red cell with eight neighbors. In this case, all neighbors are red generating a neighbor frequency of 1.0. **c** An example of a blue cell with 13 neighbors: 10 red neighbors, two blue neighbors, and one green neighbor. For the blue cell in the centre, the neighbor frequency of red cells is 0.769, the neighbor frequency of blue cells is 0.154, and the neighbor frequency of green cells is 0.077. Circles represent a fixed pixel distance from the assessed cell in each image

Cell sociology was quantified by calculating the frequency of each cell-cell interaction in each imaged area. Figure 2a shows an example where a given “red” cell has five neighbors: one “red” neighbor (1/5), one “blue” neighbor (1/5), and three “yellow” neighbors (3/5). The neighbor frequency of red cells neighboring the central red cell is 0.2, blue cells neighboring the red cell is 0.2, and yellow cells neighboring the red cell is 0.6. In contrast, Fig. 2b shows a more homogeneous example, whereby all neighbors of a given red cell are red, a neighbor frequency of 1.0. The neighbor frequency of each cell-neighbor relationship was calculated for every cell in the imaged area, and a mean neighbor frequency and standard deviation was generated for each unique cell-neighbor relationship in each area. Importantly, the mean neighbor frequency was not a function of the density of a given cell type. For example, if two rare cell types often clustered together (low cell density), the neighbor frequency would be high. In contrast to algorithms that use pixel distances from target cells to define neighbors, our method assigns neighbors as any cells that shares a Voronoi polygon border, thereby allowing cells of different sizes and distances between nuclei to be assessed as neighbors (Fig. 2a-c).

To assess the benefit of cell sociology measures to individual patients, we assessed whether individual ROIs differed from the range covered from the mean ± standard deviation of the recurrent and non-recurrent groups. The minimum and maximum values were calculated for each cell type, and for each ROI, the density and mean neighbor frequency were assessed as similar (within the range defined by the means) or different (outside the range of standard deviations). Each ROI that fell within the range by density and outside the range by cell sociology was given a + 1 value, and each ROI that was misclassified by cell sociology was given a − 1 value. Values within the range of the standard deviations were assigned a value of zero. These ROI-specific values were then summed for each patient and cell type and displayed as a heatmap (Fig. 5).

### Monte Carlo analysis of cell sociology

Monte Carlo analysis was performed on the calculated neighbor frequencies scores to determine whether these scores could be expected by random arrangements of the different cell types. The positions of all cells in the imaged area were fixed and the category of each cell position was randomized in 500 in silico simulations, while keeping the number of cells in each category fixed. This produced a histogram of neighbor frequency scores for each cell – cell interaction from the simulations, thereby allowing the calculation of z-scores for the experimental neighbor frequencies. Two hypothetical scenarios depicting a random distribution and a non-random distribution illustrate this concept (Additional file 4: Figure S4). Highly negative z-scores indicate a tendency towards avoidance while highly positive z-scores indicate a tendency towards co-localization. An absolute z-score value > 3 signifies a non-random neighbor score.

## Results

### High T cell density is associated with absence of tumor recurrence

**Fig. 3:**
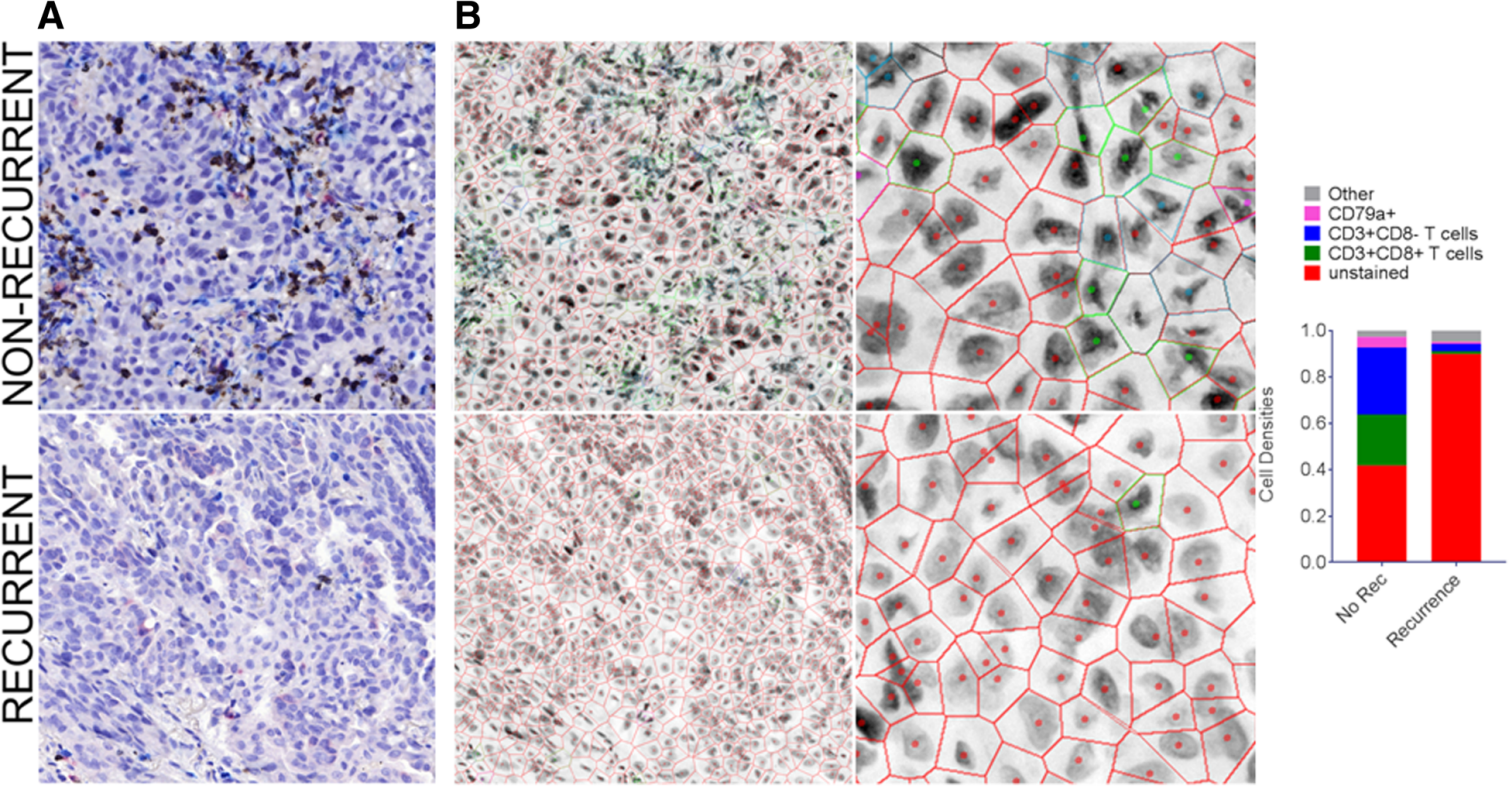
Comparison of cell densities between a recurrent and a non-recurrent lung tumor. Multiplexed immunohistochemistry staining of a non-recurrent case (top row) and a recurrent case (bottom row) of lung adenocarcinoma is shown. **a** White light imaging of the IHC stained tissue (CD3 (blue), CD8 (dark brown), CD79a (red), haematoxylin (nuclear counter stain)). **b** Voronoi diagrams of these cases are shown at equal magnification (left panels) and at increased magnification (right panels). Voronoi diagrams and cell centres are false colored red (unstained), blue (CD3+ T cells), green (CD3+ CD8+ T cells), and pink (CD79a+ B cells). (C) Cell densities of each cell types are indicated in the bar graph to the right, with “excluded” cells colored grey. These correspond to cells excluded from analysis, defined in Additional file 3: Figure S3

**Fig. 4:**
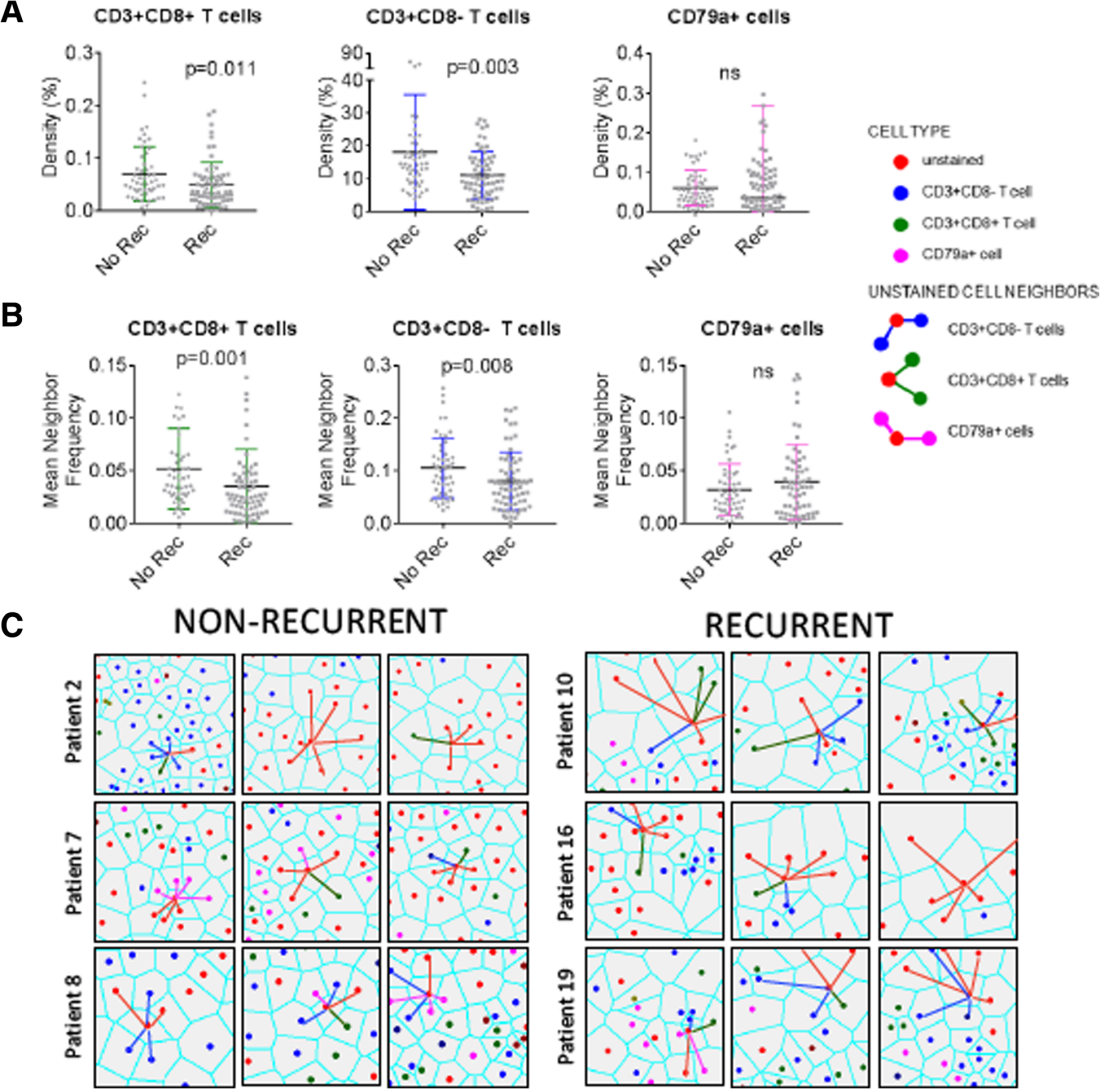
Cell densities and cell sociology of recurrent and non-recurrent lung tumors. **a** Densities of CD3+ CD8- T cells (blue), CD3+ CD8+ T cells (green), and CD79a+ cells (pink) calculated from tumor epithelial areas were compared between recurrent (*n* = 11) and non-recurrent (*n* = 8) cases of lung adenocarcinoma. **b** Cell sociology of unstained (mostly tumor) cells with CD3+ CD8- T cell neighbors (blue), CD3+ CD8+ T cell neighbors (green), and CD79a+ cell neighbors (pink) was calculated across tumor epithelial areas. The mean neighbor frequency of these interactions was compared between recurrent and non-recurrent cases of lung adenocarcinoma. In all analyses a Mann-Whitney U test *p* < 0.05 was considered significant, and bars indicate mean ± standard deviation. **c** Cell sociology of non-recurrent (left) and recurrent (right) case of lung adenocarcinoma. Each plot shows example analyses of a single unstained cell (red) within 3 fields taken from one patient slide. Cell-neighbor interactions are represented by a line color coded to match the neighbor of the unstained cell of interest. These examples highlight differences in neighbor frequencies of individual tumor cells

Increased T cell density has been associated with non-recurrence and/or survival in most tumor types analyzed to date, including lung cancer [23, 24]. To determine whether our cohort of lung adenocarcinoma samples would be sufficient to demonstrate this prognostic association, we assessed the T cell densities in our cohort of eight recurrent and 11 non-recurrent cases (Table 1). Overall survival was the only clinical variable with a statistically significant difference between recurrence groupings (*p* = 0.005). For each imaged area, we selected a sub-region that contained mostly tumor epithelium, as intraepithelial (versus intrastromal) tumor-infiltrating lymphocytes (TILs) carry the greatest prognostic significance (with implications for tumor-immune cell sociology below). Firstly, the densities of CD3+ T cells, CD3+ CD8+ T cells, and CD79a+ B cells/plasma cells were assessed. Examples of a recurrent and a non-recurrent case with low and high levels of immune cell infiltration, respectively, are shown in Fig. 3a & b. We observed significantly increased densities of both CD3+ CD8- T cells and CD3+ CD8+ T cells in non-recurrent cases of lung adenocarcinoma (*p* = 0.003 and *p* = 0.011, respectively). However, density of CD79a+ B cells was not associated with recurrence (*p* > 0.05) (Fig. 4a).

### Distinct cell sociology patterns were observed in non-recurrent cases

The primary mechanism of CD3+ CD8+ T cell activity is mediated through direct contact of the T cell receptor on the CD3+ CD8+ T cell with the MHCI/peptide/B2M complex on tumor cells, while anti-tumor activity of CD3+ CD8- T cells is mediated through both contact dependent and independent mechanisms [14]. We hypothesized that the frequency of neighboring tumor cells and T cells would be increased in non-recurrent cases. To test this hypothesis, we quantified the neighbor frequencies between unstained cells and CD3+ CD8+ T cells, unstained cells and CD3+ CD8- T cells, and unstained cells and CD79a+ B cells for all areas (*n* = 95; 8 non-recurrent and 11 recurrent patients, 5 ROIs per patient). The areas analysed were assessed by a pathologist as containing mostly tumor epithelial cells, so we considered the majority of unstained cells as tumor cells, though we could not definitively rule out some unstained stromal cell contamination.

We observed significantly higher neighbor frequencies of tumor cells with CD3+ CD8+ T cell neighbors in non-recurrent cases (*p* = 0.001, Fig. 4b, c), an association that was more significant than CD3+ CD8+ T cell density alone (*p* = 0.011, Fig. 4a, b). Figure 4c shows examples of epithelial cells interacting with other epithelial and immune cells, where the neighbor frequencies of a single tumor cell are visualized by color-coding the neighbor interactions. Even in this small sample set, these results provide intriguing preliminary data to support the improved prognostic value of cell sociology readouts over cell density alone.

Interestingly, the neighbor frequency of tumor cells with CD3+ CD8- T cell neighbors was also significantly higher in non-recurrent cases (*p* = 0.008), but the association was at a similar significance level to that observed with CD3+ CD8- T cell density alone (*p* = 0.003). The lack of increased significance is concordant with the tumor contact-independent mechanisms of anti-tumor CD3+ CD8- T cell activity [14]. Finally, we found that neither the density nor the neighbor frequency of tumor cells with CD79a+ B cell neighbors was significantly different between recurrent and non-recurrent cases, in agreement with the inconsistent prognostic significance of B cells in lung cancer [25–27] (Fig. 4a-c).

**Fig. 5:**
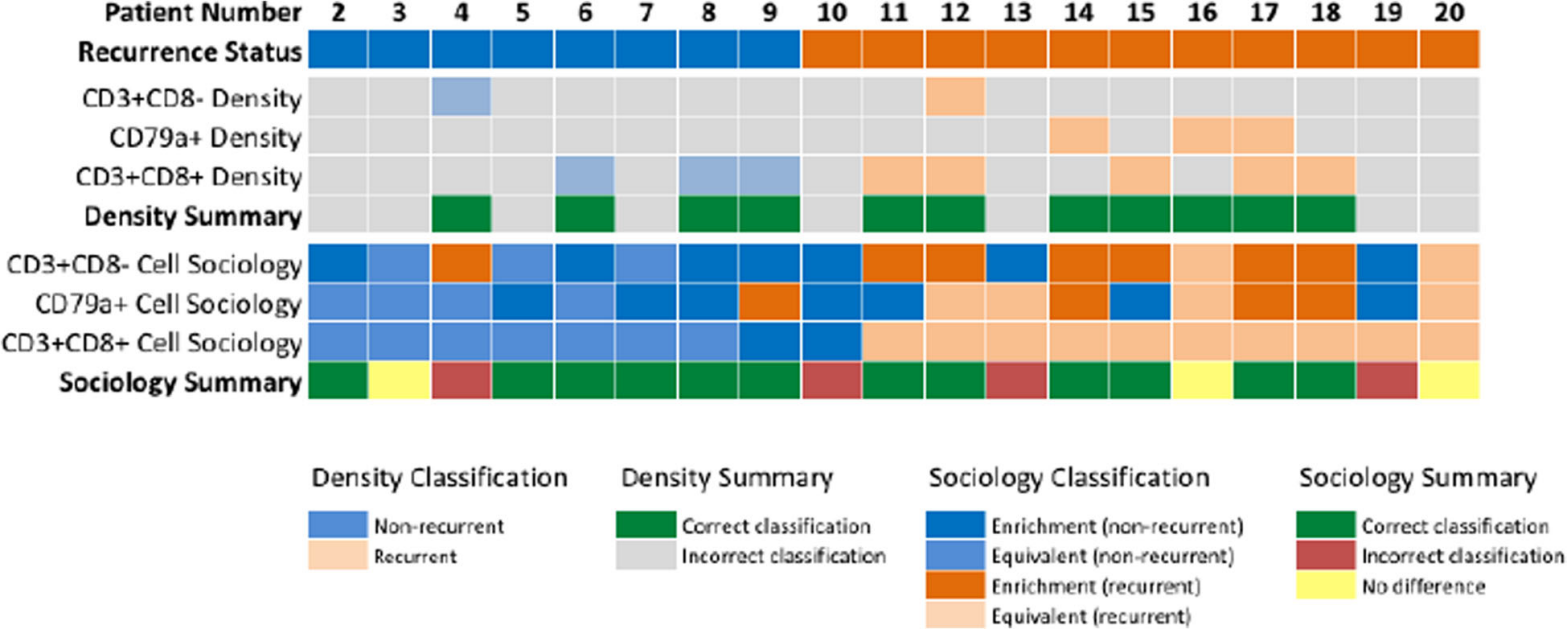
Patient-level comparison of cell sociology compared to density measurements in the prediction of recurrence. Each column represents an individual patient and recurrence status is represented as blue (non-recurrent) and orange (recurrent). The ability of cell density metrics for CD3+ CD8- T cells, CD79a+ cells, and CD3+ CD8+ T cells to correctly classify patient recurrence status by at least one ROI is represented in light blue (non-recurrent) and light orange (recurrent). Patients correctly classified by any cell density metric are indicated in green and those incorrectly classified in grey. For each patient ROI, the benefit of assessing cell sociology of tumor cells with immune cells (CD3+ CD8-, CD3+ CD8+, and CD79a) was compared to density performance. Classification enrichment in the non-recurrent direction is indicated in blue and in the recurrent direction in orange, while equivalent performance as compared to density is indicated in light blue (non-recurrent) and light orange (recurrent). Patients with an overall improvement in classification by cell sociology are indicated in green, those with no change are indicated in yellow, and those misclassified by cell sociology are indicated in red

We then assessed whether cell sociology of tumor cells with CD3+ CD8-, CD79a+, and CD3+ CD8+ immune cell neighbors was able to improve recurrence classification of individual patients as compared to the density of these three cell types. As a baseline, CD3+ CD8+ T cell density in at least one ROI was able to correctly classify eight patients; CD79a+ cell density was able to correctly classify three patients; CD3+ CD8- T cell density was able to correctly classify two patients; while eight patients were not correctly classified by any density metric. To compare, we then considered the cell sociology of these cell types with tumor cells across the ROIs. Cell sociology of CD3+ CD8+ T cells was able to perform equally as well as density in 17 patients, improve classification of one patient, and misclassified one patient. Cell sociology of CD79a+ cells was able to perform equally well as density in 8 patients, improve classification of six patients, and misclassified three patients. Cell sociology of CD3+ CD8- cells was able to perform equally as well as density in five patients, improve classification of 10 patients, and misclassified four patients. Taken together, consideration of cell sociology metrics improved classification of 12 patients as compared to density (Fig. 5). This further supports the use of cell sociology metrics as a clinically relevant read-out, which warrants further investigation in larger studies.

To assess whether the observed cell neighbor frequencies would be expected due to random distribution of the cells, we applied the Monte Carlo iterative re-sampling method. The majority of the z-scores between unstained (tumor) cells and CD3+ CD8+ T cells were highly significantly negative (z < − 3), indicating a non-random distribution of cells and a tendency towards avoidance (Additional file 5: Figure S5). This suggests that the increased proximity of tumor cells and CD3+ CD8+ T cells observed in non-recurrent cases is indicative of a biologically relevant immune process associated with disease recurrence. Thus, our pilot study demonstrates the promise of utilizing tumor and immune cell sociology features to improve prognostic accuracy.

## Discussion

**Fig. 6:**
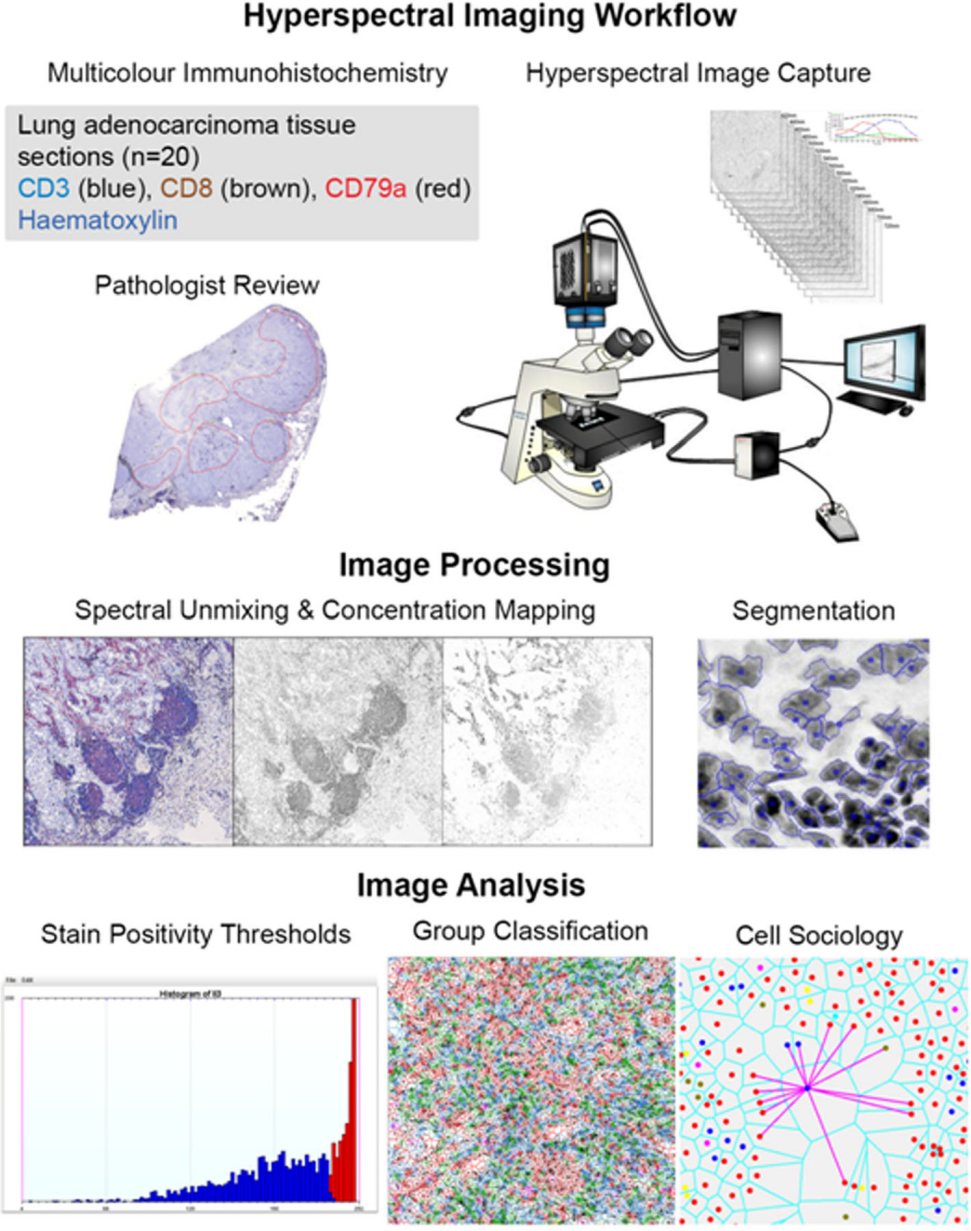
Hypserspectral imaging workflow. Firstly, the tissue was stained with an optimized multiplexed antibody panel. The staining was then assessed by a pathologist and regions of interest were identified for image acquisition. Hyperspectral images corresponding to five areas per tissue slide were then captured. Acquired images underwent spectral unmixing and mapping of unmixed stain concentrations. Images were then segmented using nuclear stain characteristics. Segmented images were further analyzed to determine stain positivity thresholds, and based on stain positivity and negativity, cells were classified into groups. Finally, the spatial relationships of all groups were interrogated and quantified

The quantification of cell-cell interactions and spatial relationships within the tumor architecture is likely to provide more precise biological information compared to cell densities alone, and holds as-of-yet unknown clinical potential. We have developed a novel pipeline for hyperspectral imaging and cell sociology analysis of multiplexed IHC specimens (Fig. 6), and applied it to 100 areas from 20 FFPE tumor sections of lung adenocarcinoma (CD3, CD8, CD79a, haematoxylin). The quantification of cell-cell spatial relationships within the TME allowed a more comprehensive examination of immune-tumor cell interactions. Our cell sociology analysis revealed that the spatial organization of CD3+ CD8+ T cells within the tumor epithelium may provide additional prognostic value compared to CD3+ CD8+ T cell density alone. This research provides a framework for future studies investigating how the spatial context of diverse cell types in the TME could reveal new insights for both biological and clinical studies.

The analysis of tumor architecture and spatial organization of cell-cell interactions in histology specimens is challenging. While two other systems exist to identify neighborhoods, they both rely primarily on distance between nuclei [28, 29]. Our system uses Voronoi tessellation, which has the advantage of not requiring arbritrary definitions of neighborhoods (Fig. 2). Since each Voronoi polygon is interpreted as an approximation of the cell boundary, only cells contacting each other (sharing common Voronoi edge) are considered neighbors. This approach accounts for the natural variability in tissue architecture in which cell neighbors can be observed to be closely packed (e.g. clusters of small immune cells) or distant (e.g. disorganized tumor cells) from one another (Fig. 2). Despite using different neighborhood concepts, these technologies should provide similar results, though discrepancies could arise when analyzing low frequency cell populations. Further studies are required to compare and validate experimental data generated by these different technologies.

As the complexity of staining panels increases in the quest to improve resolution of cell types, so does the importance of statistical methods to ensure the correct interpretation of results. We incorporated the Monte Carlo iterative re-sampling methodology to provide a z-score indicating that a cell-cell interaction was increased or decreased more than random distribution would anticiapte. Indeed, our results demonstrate that the spatial organization of different cell subsets is highly non-random, likely indicating underlying biological phenotypes. The use of these and other statistical methods are becoming increasingly important as technology improvements allow for the analysis of whole slide images, that consist of greatly increased cell numbers and exponentially increased cell sociology.

The investigation of the prognostic value of immune cell infiltrate in tumors has gained much interest over the last decade, and several new immunotherapies have been approved for treatment of a variety of cancer types [30]. For example in colorectal cancer, the density of CD8+ T cells at the tumor invasive margin (dubbed the “immunoscore”) is more predictive of outcomes compared to the traditional Tumor; Node; Metastasis (TNM) scoring [6] or microsatellite instability scores [31, 32]. Our methodology may be used to augment the prognostic value of the immune infiltrate. Despite the limited sample size of our pilot study (*n* = 20), we demonstrate that the spatial organization of CD3+ CD8+ T cells and unstained cells in tumor epithelial areas is more significantly associated with recurrence than CD3+ CD8+ T lymphocyte density alone. On the patient level, we also find that cell sociology improves the classification of 6/8 non-recurrent and 6/11 recurrent patients when compared to classification by cell density. This reinforces our contention that the addition of cell sociology quantifications to currently available prognostic or predictive tools would add advance our insight into anti-tumor immunity. Furthermore, our technology could be used for investigating immune phenotypes in patients receiving immunotherapy. For example, the use of PD-L1 expression as a biomarker for anti-PD1 immunotherapy response is currently under investigation [33–35], but major challenges have arisen due to staining heterogeneity [10, 11]. PD-L1 may be expressed as a continuum ranging from low to high levels on tumor cells, and on tumor-associated immune cells (macrophages, being a relevant population in this regard). Thresholds for treatment are based on combinations of positive tumor cells, and sometimes immune cells, and cut-off points in the treatment algorithm vary with different analytical IHC antibody clones and detection platforms and remain a source of uncertainty for pathologists and oncologists involved in lung cancer [36]. Our cell sociology approach could conceivably identify specific spatial relationships between T cells, other immune cells, and PD-L1 expressing cells with the intention of refining checkpoint blockade immune oncology treatments.

Hyperspectral Cell Sociology analysis revealed patterns congruent with our current understanding of anti-tumor immunity. For example, an increased frequency of CD3+ CD8+ T cells neighboring unstained cells (predominantly tumor epithelial cells but also stromal cells) was highly associated with non-recurrence. This morphological observation correlated with the known biological mechanism of action of cytotoxic T cells, which exert their tumor cell killing function through direct cell-to-cell contact. Indeed, in our analysis, there was no association between absence of recurrence and any immune cell type neighboring any other immune cell type (CD3+ CD8+ − T cells, CD3+ CD8- T cells, CD79 + -B cells; data not shown). These data suggest that clusters of immune cells – typically observed in fibrovascular stromal areas juxtaposed to tumor epithelium of certain lung cancers – may be less important to tumor rejection than dispersed within the tumor epithelium. Additionally, we found that CD3+ CD8- helper T cells were associated with absence of tumor recurrence, but at a similar level as the CD3+ CD8- T cell density alone. CD3+ CD8- T cells can exert their effects by expressing immune activating (or inhibiting) cytokines, expressing growth inhibitory cytokines, killing target cells, and/or increasing the activity of other T cells and B cells [14]. These mechanisms may have actions on cells more distant than the nearest neighbor of the T cell. To address this, the relationship of 2nd and 3rd order neighbors (ie. cells 2 and 3 cells away from a given cell) on tumor behavior is an avenue for future investigation. Finally, we found that cell sociology between B cells and tumor cells was not associated with recurrence. Since antibodies produced by B cells circulate systemically, direct interactions of B cells and tumor cells may not be necessary for anti-tumor B cell activity. Analysis of a larger cohort of lung cancer patients would be required to resolve the more subtle influences that B cells may have on the TME.

## Conclusions

We have developed a pipeline for the analysis of cell sociology from hyperspectral IHC images in lung cancer. We believe future studies quantifying cell-cell spatial interactions will generate enhanced biological insight not currently available from cell density information alone. The study of cell sociology could be applied to any number of scenarios such as interactions between immune cells and tumor cells, different subclonal tumor cells, and tumor cells and stromal cells. Cell sociology has broad cross-dispiplinary applications, and is powerful tool for the understanding of biological events in which cell-cell spatial interactions dictate functionality, including cancer immunology.

## Additional files

Additional file 1: **Figure S1.** Spectral unmixing. An example of a region of interest captured by white-light imaging (top left). The subsequent panels represent the concentration maps of the spectrally unmixed hyperspectral image for all spectra of interest: haematoxylin, CD79a (red), CD3 (blue), CD8 (brown), and black artifacts. (TIF 1141 kb)

Additional file 2: **Figure S2.** Histogram of the integrated intensity values of a given stain. The histogram represents the number of segmented cells (y-axis) as a function of integrated stain intensity (II3) (x-axis, max = 0, min = 255). In this example, cells below a threshold of 234 are considered positively stained (blue), while those above a threshold of 234 are negative (red). (TIF 102 kb)

Additional file 3: **Figure S3.** Cell categorization into groups based on stain characteristics. (A) A binary decision tree was used to catetgorize all segmented cells into groups. Cells positive for incompatible lineage markers (CD79a+/CD3+ or CD79a+/CD3+/CD8+) required reassignment, whereas CD8+ CD3- and CD8+ CD79a+ cells were rare and excluded from analysis. (B) Cells positive for incompatible markers were visually assessed for evidence of cell overlap occurring as a result of improper segmentation due to object proximity. In this example of a CD3+ CD79a+ cell, the topmost image shows CD3+ (blue) and CD79a+ (red) channels visualized in false color simultaneously and the nuclear boundary is shown as a red line. The second image shows only the CD3+ channel. The third image shows only the the CD79a+ channel. Since the presence of two overlapping cells is clear, a second nuclear centre is produced and the CD3+ CD79a+ cell is reassigned to a CD3+ cell immediately adjacent to a CD79a+ cell (bottom image). (C) In this example of a CD3+ CD79a+ cell, there is not clear evidence of two adjacent cells. As double positivity for these markers is not supported by current literature, these cells were rare and excluded from analysis. (TIF 207 kb)

Additional file 4: **Figure S4.** Monte Carlo simulation. (A) Hypothetical samples depicting a random distribution (sample 1) and a non-random distribution (sample 2) are shown and colors represent different phenotypes of cells. (B) The neighbor score (top) and z-scores (bottom) of each combination of nearest neighbor interactions are shown. Low neighbor frequency of red cells with blue cell neighbors were present in sample 1; furthermore, the z-score of this observation was near zero, indicating this interaction would be expected from random (non-meaningful) distributions of the cells. In contrast, in sample 2, the neighbor frequency of red to blue cells was 0.6 and the z-score value was 24. The interactions happened much more frequently than would be expected by random distribution of the cells, meaning experimental data matching this pattern may indicate an underlying biological phenotype. (TIF 653 kb)

Additional file 5: **Figure S5.** Monte Carlo z-scores of mean neighbor frequencies. Histograms of cell sociology z-scores generated by Monte Carlo analysis of unstained (tumor) cells with CD3+ CD8+ T cell neighbors in non-recurrent (top) and recurrent (bottom) cases. Cutoff z-scores of +/− 3 were used to assess whether the distributions were likely to be non-random; the highly negative scores represent a tendency towards avoidance. (TIF 122 kb)

## Abbreviations

B2M: Beta 2 Microglobulin
BCCA: British Columbia Cancer Agency
FFPE: Formalin fixed paraffin embedded
IHC: Immunohistochemistry
MHCI: Major Histocompatibility Complex I
PD-1: Programmed cell Death 1
PD-L1: Programmed cell Death Ligand 1
ROI: Regions Of Interest
TNM: Tumor; Node; Metastasis
