# Supplementary figures and images for: Hyperspectral cell sociology reveals spatial tumor-immune cell interactions associated with lung cancer recurrence
Source: J Immunother Cancer. 2019 Jan 16;7:13. doi: 10.1186/s40425-018-0488-6 (PMC6335759; doi:10.1186/s40425-018-0488-6)

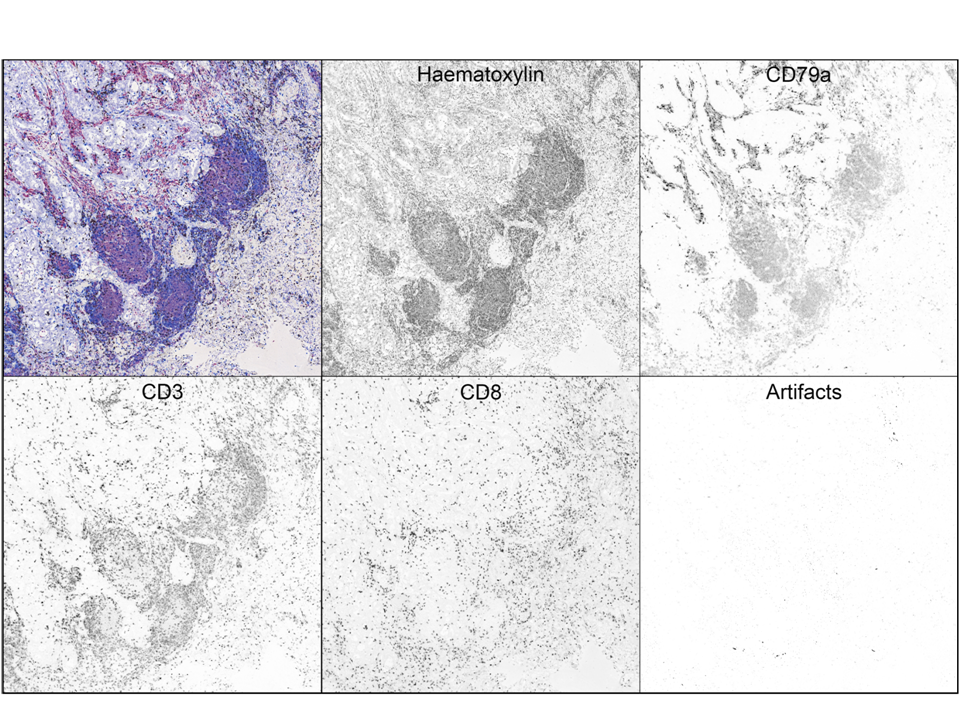

Supplement: Supplementary file 1 — Figure S1. Spectral unmixing. An example of a region of interest captured by white-light imaging (top left). The subsequent panels represent the concentration maps of the spectrally unmixed hyperspectral image for all spectra of interest: haematoxylin, CD79a (red), CD3 (blue), CD8 (brown), and black artifacts. (TIF 1141 kb) [file 40425_2018_488_MOESM1_ESM.tif]

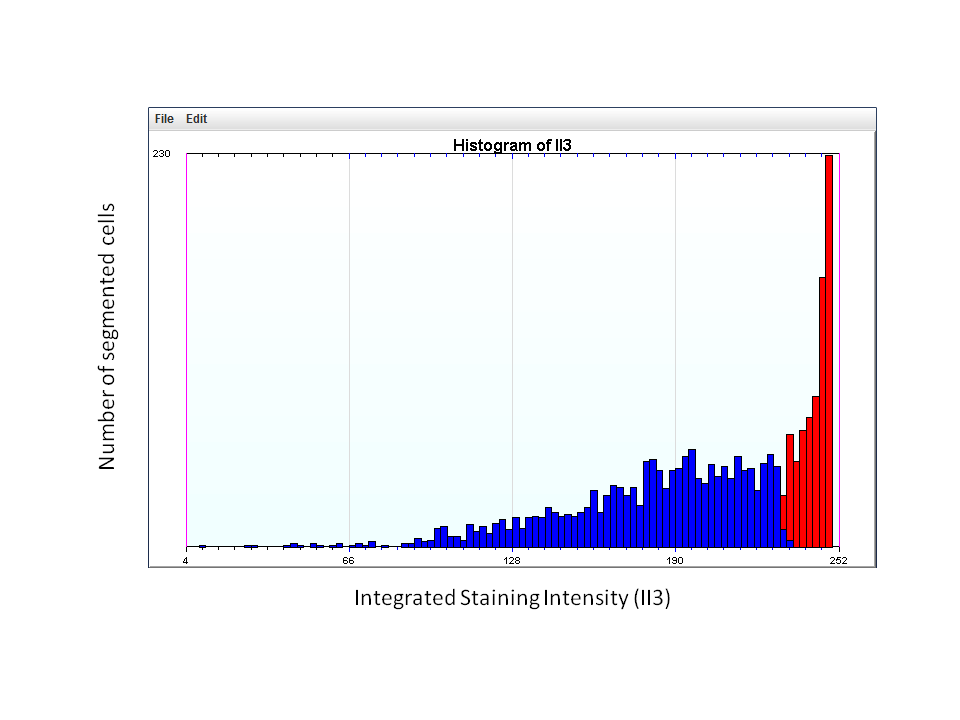

Supplement: Supplementary file 2 — Figure S2. Histogram of the integrated intensity values of a given stain. The histogram represents the number of segmented cells (y-axis) as a function of integrated stain intensity (II3) (x-axis, max = 0, min = 255). In this example, cells below a threshold of 234 are considered positively stained (blue), while those above a threshold of 234 are negative (red). (TIF 102 kb) [file 40425_2018_488_MOESM2_ESM.tif]

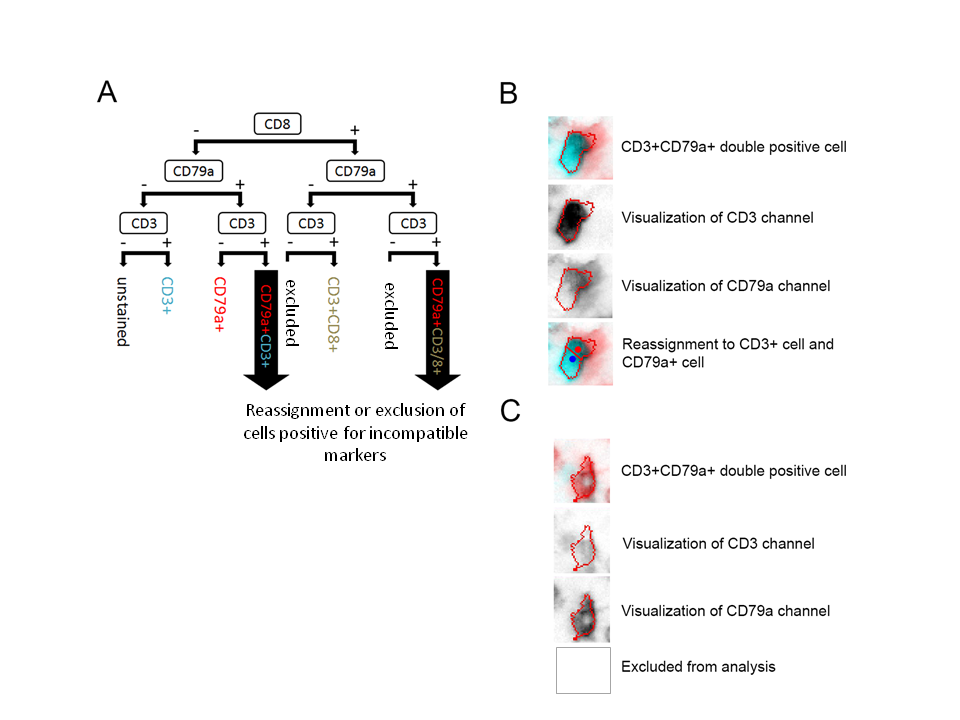

Supplement: Supplementary file 3 — Figure S3. Cell categorization into groups based on stain characteristics. (A) A binary decision tree was used to catetgorize all segmented cells into groups. Cells positive for incompatible lineage markers (CD79a+/CD3+ or CD79a+/CD3+/CD8+) required reassignment, whereas CD8+ CD3- and CD8+ CD79a+ cells were rare and excluded from analysis. (B) Cells positive for incompatible markers were visually assessed for evidence of cell overlap occurring as a result of improper segmentation due to object proximity. In this example of a CD3+ CD79a+ cell, the topmost image shows CD3+ (blue) and CD79a+ (red) channels visualized in false color simultaneously and the nuclear boundary is shown as a red line. The second image shows only the CD3+ channel. The third image shows only the the CD79a+ channel. Since the presence of two overlapping cells is clear, a second nuclear centre is produced and the CD3+ CD79a+ cell is reassigned to a CD3+ cell immediately adjacent to a CD79a+ cell (bottom image). (C) In this example of a CD3+ CD79a+ cell, there is not clear evidence of two adjacent cells. As double positivity for these markers is not supported by current literature, these cells were rare and excluded from analysis. (TIF 207 kb) [file 40425_2018_488_MOESM3_ESM.tif]

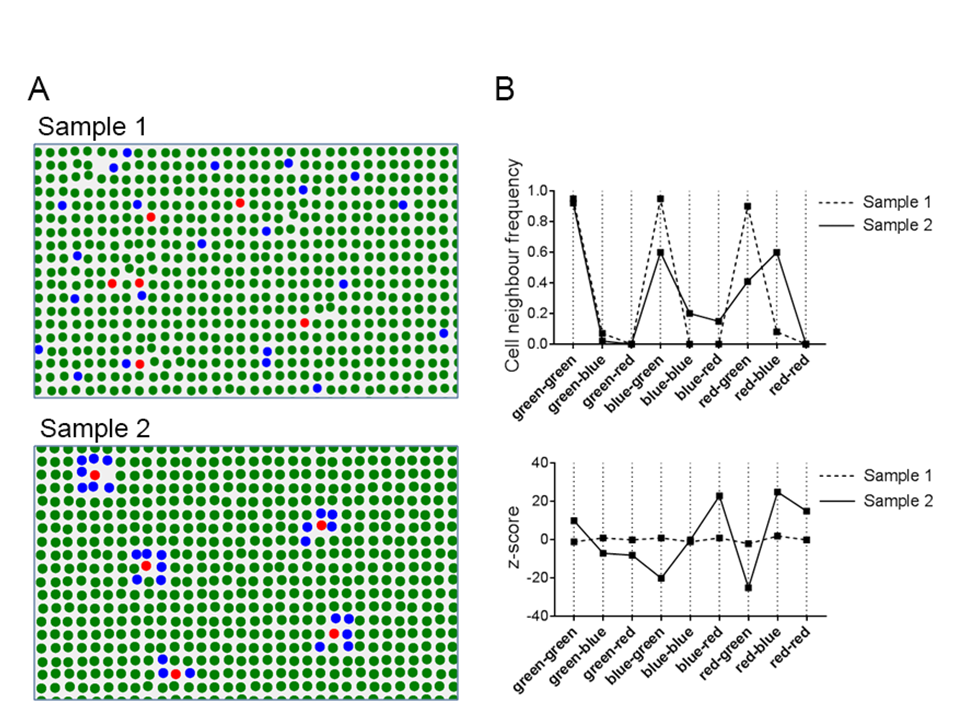

Supplement: Supplementary file 4 — Figure S4. Monte Carlo simulation. (A) Hypothetical samples depicting a random distribution (sample 1) and a non-random distribution (sample 2) are shown and colors represent different phenotypes of cells. (B) The neighbor score (top) and z-scores (bottom) of each combination of nearest neighbor interactions are shown. Low neighbor frequency of red cells with blue cell neighbors were present in sample 1; furthermore, the z-score of this observation was near zero, indicating this interaction would be expected from random (non-meaningful) distributions of the cells. In contrast, in sample 2, the neighbor frequency of red to blue cells was 0.6 and the z-score value was 24. The interactions happened much more frequently than would be expected by random distribution of the cells, meaning experimental data matching this pattern may indicate an underlying biological phenotype. (TIF 653 kb) [file 40425_2018_488_MOESM4_ESM.tif]

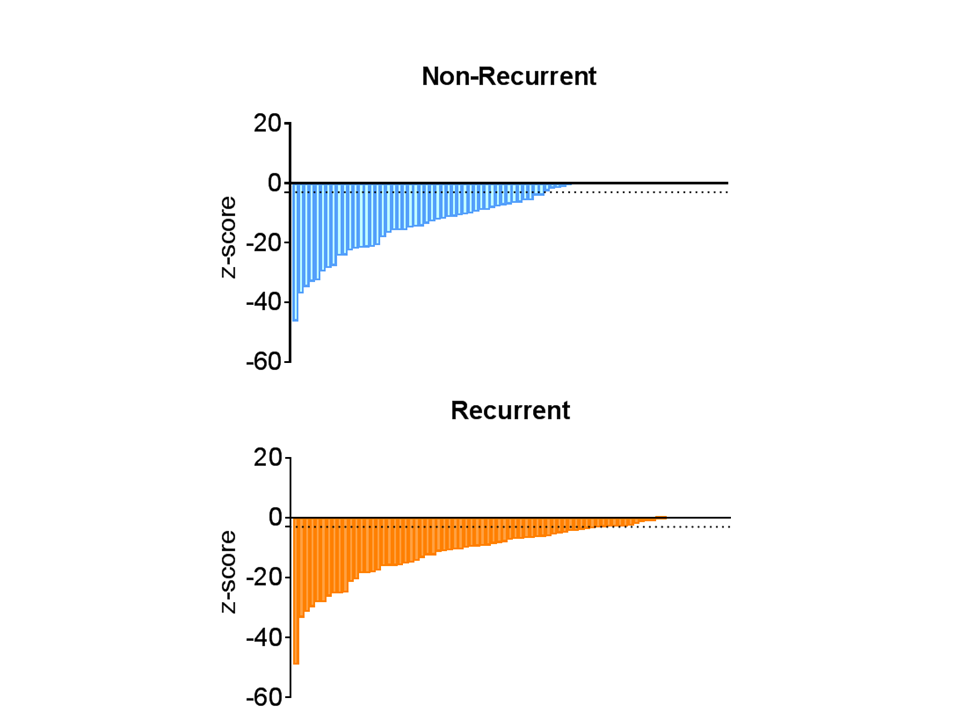

Supplement: Supplementary file 5 — Figure S5. Monte Carlo z-scores of mean neighbor frequencies. Histograms of cell sociology z-scores generated by Monte Carlo analysis of unstained (tumor) cells with CD3+ CD8+ T cell neighbors in non-recurrent (top) and recurrent (bottom) cases. Cutoff z-scores of +/− 3 were used to assess whether the distributions were likely to be non-random; the highly negative scores represent a tendency towards avoidance. (TIF 122 kb) [file 40425_2018_488_MOESM5_ESM.tif]
